# Supplementary material for: SARS-CoV-2 seroprevalence study in Lambayeque, Peru. June–July 2020
Source: PeerJ. 2021 Apr 6;9:e11210. doi: 10.7717/peerj.11210 (PMC8034367; doi:10.7717/peerj.11210)
Supplement: Supplemental Information 2 — COPD: Chronic obstructive pulmonary disease. ARI: Acute respiratory infection [file peerj-09-11210-s002.docx]

**SARS-CoV-2 seroprevalence study in Lambayeque, Peru. June-July 2020**

| **Variable**  (Variable Name) | **Codebooks** | **Reference Group**  Omitted in the regression model |
| --- | --- | --- |
| Inhabitants | 1: < 20000 hab.  2: 20000-50000 hab.  3: >50000 hab | - |
| Syntomatic | 0: No  1: Yes | - |
| Comorbility | 0: No  1: Yes | - |
| Seroprevalence | 0: No  1: Yes | No |
| Isolation | 0: No  1: Yes | No |
| Fever/chills | 0: No  1: Yes | No |
| General malaise | 0: No  1: Yes | No |
| Cough | 0: No  1: Yes | No |
| Sore throat | 0: No  1: Yes | No |
| Nasal congestion | 0: No  1: Yes | No |
| Shortness_breath | 0: No  1: Yes | No |
| Diarrhea | 0: No  1: Yes | No |
| Nausea/vomiting | 0: No  1: Yes | No |
| Headache | 0: No  1: Yes | No |
| Irritability/confusion | 0: No  1: Yes | No |
| Dysosmia | 0: No  1: Yes | No |
| Dysgeusia | 0: No  1: Yes | No |
| Muscle pain | 0: No  1: Yes | No |
| Abdominal pain | 0: No  1: Yes | No |
| Chest pain | 0: No  1: Yes | No |
| Backache | 0: No  1: Yes | No |
| Pregnancy | 0: No  1: Yes | No |
| Hypertension | 0: No  1: Yes | No |
| Diabetes | 0: No  1: Yes | No |
| Liver diseases | 0: No  1: Yes | No |
| Renal diseases | 0: No  1: Yes | No |
| COPD | 0: No  1: Yes | No |
| Cancer | 0: No  1: Yes | No |
| Obesity | 0: No  1: Yes | No |
| Travel | 0: No  1: Yes | No |
| Previous_visit_healthcare_facility | 0: No  1: Yes | No |
| Contact_ARI_case | 0: No  1: Yes | No |
| conctaco_confirmed_case_covid | 0: No  1: Yes | No |
| visit_market_14days_before | 0: No  1: Yes | No |
